# Using shear-wave elastography in skeletal muscle: A repeatability and reproducibility study on biceps femoris muscle

**DOI:** 10.1371/journal.pone.0222008

**Published:** 2019-08-30

**Authors:** Nejc Šarabon, Žiga Kozinc, Nastja Podrekar

**Affiliations:** 1 University of Primorska, Faculty of Health Sciences, Izola, Slovenia; 2 University of Primorska, Andrej Marušič Institute, Koper, Slovenia; 3 InnoRennew Center of Excellence, Izola, Slovenia; Universitat Zurich, SWITZERLAND

## Abstract

Shear-wave electrography (SWE) is a method used to assess tissue elasticity. Recently, it has been used to assess muscle stiffness, but the reliability of SWE for this purpose has not been thoroughly investigated. The purpose of this study was to evaluate the repeatability and reproducibility of SWE on porcine meat specimens and the human biceps femoris muscle. Measurements on meat specimens (n = 20) were performed by three raters and with a custom-built device that allowed constant application force. Measurements on human participants (n = 20) were performed by two raters in relaxed and stretched muscle positions on two visits. Most aspects of repeatability and reproducibility were good or high, with intra-class correlation coefficient (ICC) values above 0.70. Minimal detectable changes were lower in a relaxed (6–10%) than stretched (15-16%) muscle position. In conclusion, SWE is a reliable tool for assessing muscle stiffness if the muscle is examined in relaxed condition, while changing the force applied with the probe for as little as 1.5 N results in significantly lower repeatability.

## Introduction

Ultrasound imaging plays a major role in the diagnosis and monitoring of several diseases. Modern ultrasound devices do not only allow clinicians to see internal organs, but also to assess the mechanical properties of various tissues without palpation. In past two decades, there have been significant advances in ultrasound methods for measuring mechanical elasticity [1–3]. In particular, shear-wave elastography (SWE) emerged about 20 years ago [3,4] and has been developing rapidly since. In brief, this method is based on evaluating shear wave propagation speed. The probe generates a radiation force in tissue which creates a shear wave. The wave then propagates and is captured by taking consecutive ultrasound images at a high repetition frequency [4–6]. Next, the shear modulus (μ) is calculated as follows:

$$\mu =\rho \times c^{2}$$

where ρ is the density of the tissue and c is the shear wave propagation speed. SWE is appreciated as fast, non-invasive and easy-to-use [5]. In addition to qualitative assessment (real-time visual feedback on elasticity), state of the art devices also allow quantification of elasticity in selected region of interest. Several papers have reported good diagnostic performance of SWE in medicine, including diagnosing of breast lesions [7], cervical lymph nodes [8], thyroid nodes [9], liver fibrosis [10] and pancreas abnormalities [11].

In recent years, the benefits of using SWE have expanded beyond diagnostics in medicine. For instance, SWE has been used to assess muscle stiffness [12–17], which is an important factor in physical function, movement execution and performance in sport. Moreover, SWE is a useful tool to evaluate changes of muscle properties in observational and experimental studies. Using SWE, it has been shown that muscle stiffness is influenced by age [18,19], sex [19] and different neuromuscular diseases, such as cerebral palsy [12] and Parkinson’s disease [14]. Furthermore, shear modulus values are significantly affected by joint positions and consequent changes in passive muscle stiffness [20–22]. Acute effects of stretching [15,20,23], warm-up protocols [24] and muscle contraction [25] have also been demonstrated. Practitioners should be aware of the importance of technical settings, particularly the size and depth of the region of interest [26,27].

Repeatability and reproducibility of SWE to quantitatively assess stiffness of skeletal muscle have been investigated by several authors, beginning on meat specimens [13] and continuing with several human experiments [28–32]. While the results mostly reveal good repeatability and reproducibility of SWE, the majority of studies to date did not investigate all aspects of reliability (most often neglecting inter-visit reproducibility) and focused mostly on trunk [30,32] and shoulder region [29]. On the other hand, investigating lower leg muscles is as important in many fields, such as injury prevention, strength and conditioning, and physical therapy. The biceps femoris (BF) muscle has been of particular interest in sports medicine research. However, knowledge on the reliability of BF muscle stiffness assessment using SWE is limited [20,28]. Furthermore, the sources of measurement errors, however small, have not been clearly identified. One of our aims was to assess what are the potential sources of errors (e.g. ultrasound device itself, human errors, or the dynamism of the muscle tissue). Therefore, the purpose of this study was to assess intra-rater repeatability and inter-rater and inter-visit reproducibility of SWE-derived shear modulus: A) *ex vivo* (using thigh porcine meat specimens), B) *in vivo* on relaxed human BF muscle, C) *in vivo* on stretched human muscle and D) using different levels of force applied by the probe. We hypothesized that SWE will have good to excellent repeatability and reproducibility in all measured conditions.

## Methods

### Participants

Twenty healthy participants (8 males, 12 females; age: 31.4 ± 9.8 years; height: 171.7 ± 10.4 cm; body mass: 69.0 ± 12.8 kg) volunteered for the study. The inclusion criteria were absence of lower limb injuries and absence of chronic diseases or any current neuromuscular problems. Before the measurements, participants were informed about the aims and procedures of the study and were required to sign an informed consent form to participate. The study was approved by National Medical Ethics Committee of Slovenia (reg. number 0120-690/2017/8).

### Meat specimens

For the first part of the experiment, five fresh porcine thigh meat specimens were obtained from a local meat distributor. Four distinct separate points were selected and marked on each specimen for a total sample of 20 points. Typical size of the meat samples was 15 × 15 × 10 cm. Meat specimens were kept at room temperature for 3 hours before the measurements begun, to ensure that the tissue temperature was stable and did not affect shear modulus values.

### Study design and procedures

All the examiners in our study were briefly (2 sessions of 1 hour) trained by an expert in ultrasound imaging. These sessions included training of the probe positioning and discerning the different anatomical structures. All examiners had at least a master’s degree in kinesiology and were familiar with the anatomical details of the musculoskeletal system of the lower limb.

**Fig 1:**
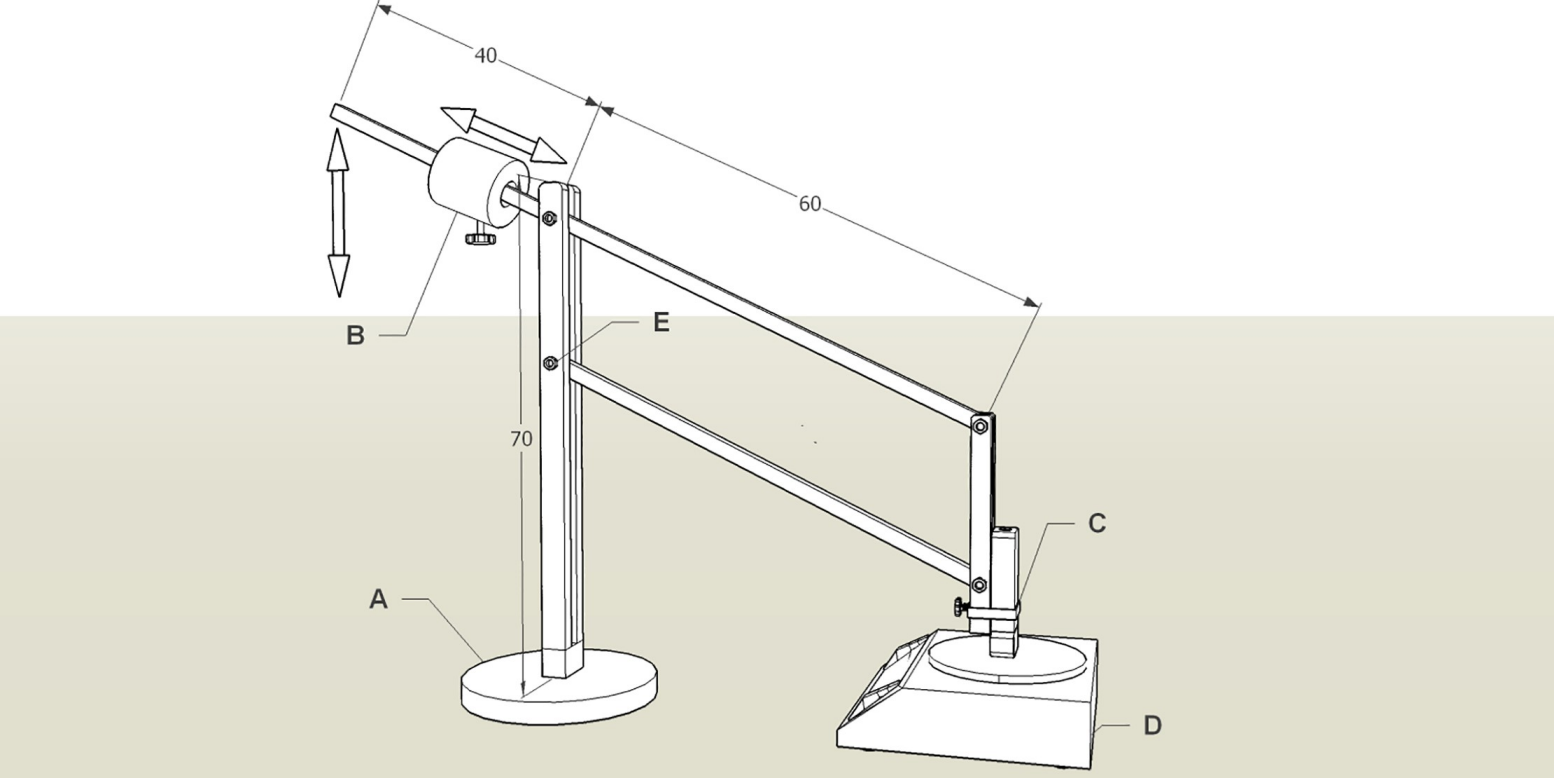
The CFAD was custom-made out of spruce wood. It consists of solid metal base (A), the wooden pylon, and horizontal swing-like handle. On one side of the handle, an iron weight (1200 g) is placed (B). The weight can be moved along the handle in order to change the required force application. The probe is fixed to the device with an adjustable plastic cluster (C). The positions of the weight corresponding to a desired force levels were determined and regularly checked by applying the load on a digital strain gauge-based scale (D). Ball-bearings (E) were used to connect the pylon with the swing.

During the measurements, the examiners were blinded to the results of each other until the end of the experiments, and were not allowed to be present in the same room during measurements of the other examiners. For the meat specimen experiment, three examiners performed the measurement at each point three times, allowing intra-rater repeatability and inter-rater reproducibility evaluation (Table 1, Part 1). Inter-visit reproducibility was not assessed for meat specimens. Additionally, each point was assessed three times using a custom-built constant force application device (CFAD) (Fig 1) with two different levels of force application (Table 1, Part 2). A similar approach has been used before to ensure inter-visit reproducibility of the SWE measurements [33]. Low- and high-force levels were set at 2.5 and 4 N, respectively, and the accuracy was regularly checked by applying the force to the digital strain gauge-based scale, placed on the same height as the skin of the participants. The CFAD was always operated by the same examiner. These measurements were conducted to separately assess the repeatability of SWE when using exact same probe position and orientation, and exact same or different application force. Deviations from desired values of force never exceeded 0.1 N (10 g). The CFAD also allowed the operator to keep the probe orientation (longitudinal to the muscle fibres for human experiments) and position (perpendicular to the skin) constant. The examiners were instructed to keep the probe perpendicular to the skin for their assessment.

**Table 1:** Overview of steps undertaken in the experiment.

Part	Raters	Tissue	Repetitions	Analysis
1	Human	Specimen	3 repetitions done by each of the 3 raters (force 4 N).	Intra-rater repeatability and inter-rater reproducibility on a stable tissue
2	CFAD	Specimen	3 repetitions with low force (2.5 N) 3 repetitions with high force (4 N)	Repeatability with stable force, tissue and position conditions; Effects of changing the application force;
3	CFAD	Human (relaxed)	3 repetitions with high force* (force 4 N)	Repeatability in stable force and position conditions *in vivo*.
4	Human	Human (relaxed)	3 repetitions done by each of the 2 raters* (force 4 N)	Intra-rater repeatability and inter-rater-reproducibility *in vivo*.
5	Human	Human (stretched)	3 repetitions done by each of the 2 raters* (force 4 N)	Intra-rater repeatability and inter-rater-reproducibility *in vivo* on a stretched muscle tissue.

* performed additionally on a separate visit to analyze inter-visit reproducibility

For the *in vivo* experiments, the lateral part of the hamstring muscle (i.e., the long head of the BF muscle) on the kicking dominant leg of each participant was assessed. The location of the measurement was set at 50% on the line between the lateral epicondyle of the tibia and the ischial tuberosity, with the probe positioned longitudinally to the muscle fibre. First, two examiners each performed three repetitions in a relaxed position (Table 1, Part 3), followed by additional three repetitions using the CFAD (Table 1, Part 4).

Next, each of the two examiners performed an additional three measurements at the BF muscle of the tested leg while it was stretched. On a second visit, which was 2–4 days after the first visit, the measurements were repeated. For the assessment in a relaxed position, participants laid prone, with the thigh slightly elevated to ensure that the probe was pressed perpendicularly to the skin. Assessment of maximal hip flexion angle and maintaining standardized stretch (60% of the maximal hip flexion angle at full knee extension) was achieved using HUMAC NORM Isokinetic dynamometer (Computer Sports Medicine Inc., Stoughton, MA, USA) in passive mode. Participants lay supine and the examined leg was slowly lifted by the examiner, and range of motion was set determined when first sign of pain was reported. The target position for SWE measurements was maintained by locking at the appropriate angle. Between repetitions, the leg was lowered to neutral position. Muscle tissue is well reported to be highly anisotropic [31,32], resulting in different shear-wave speeds when probe orientation relative to the fibre is changed. Therefore, longitudinal alignment to the muscle fibres was used for both meat specimen and human tissue experiments.

### Equipment and data acquisition

A diagnostic ultrasound system, Resona 7 (Mindray, Shenzhen, China), was used for all measurements. Muscle stiffness was assessed by acquiring shear modulus (kPa) data. The ultrasound system was set to musculoskeletal SWE mode (assuming tissue density of 1000 kg/m^3^) and middle-sized linear probe (Model L11-3U, Mindray, Shenzhen, China) with water-soluble, hypoallergenic ultrasound gel (AquaUltra Basic, Ultragel, Budapest, Hungary) was used. For each single repetition, the mean value of eight quick consecutive scans (duration: <10 s) was calculated.

**Fig 2:**
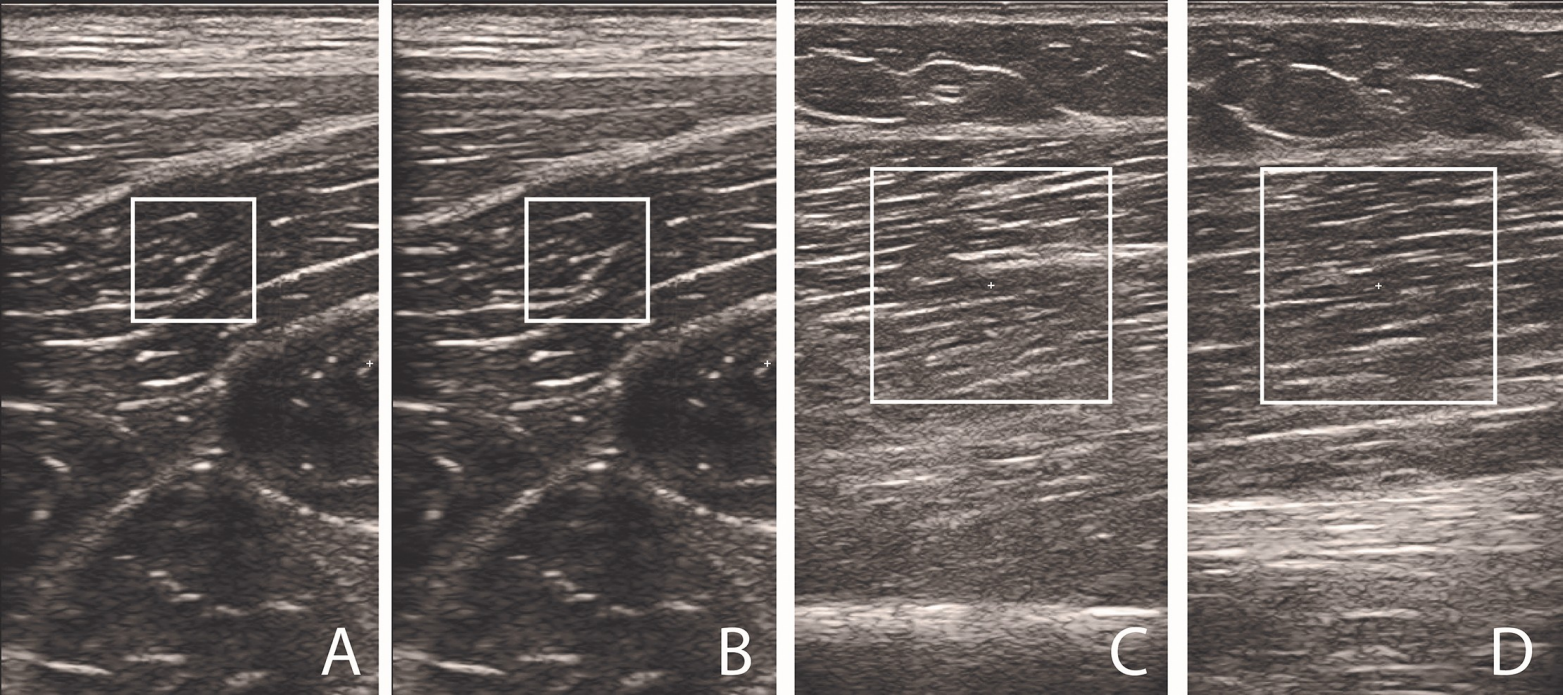
Examples of typical region of interest positioning. Low and high force application conditions resulted in minimal (i.e. visually undetectable) changes in the image (A and B). Images C and D show examples of region of interest positioning on a selected human participant for relaxed and stretched condition, respectively.

The size of the region of interest was set to 1 × 1 cm for meat specimen measurements and 2 × 2 cm for all measurements on human participants. The depth of region of interest was chosen based on the specimens’ or participants’ anthropometric characteristics in a way that the whole region was targeting muscle tissue. Examples of the region of interest positioning on a human BF muscle are presented in Fig 2.

### Statistical analysis

Statistical analysis was performed using SPSS (version 20.0, SPSS Inc., Chicago, USA). Descriptive statistics were calculated and reported as the mean ± standard deviation. Pearson correlation coefficients were calculated to assess the associations between region of interest depth and shear modulus values. Repeatability and reproducibility were assessed using two-way mixed single (ICCs) and average (ICCa) intra-class correlation coefficients. ICC scores were interpreted as: fair (ICC 0.40–0.59); moderate (ICC 0.60–0.74) and good to excellent (ICC 0.75–1.00)[34]. For the intra-rater repeatability ICC, we used the data of one examiner only. Within-subject variation was assessed using standard error of measurement (SEM) and coefficients of variation (CV% = SEM/mean × 100). Minimal detectable change (MDC) was calculated (MDC = SEM × 1.96 × √2) to determine the magnitude of change in shear modulus score that would exceed the threshold of measurement error at the 95% confidence level. To check for a systematic bias between the trials, repeated measures analysis of variance (RM ANOVA) was used. A paired-sample t-test was used to evaluate the differences between SWE scores in relaxed and stretched muscle. The level of significance was set to p < 0.05 for all analyses.

## Results

Mean depth of the centre of the region of interest was 2.32 ± 0.37 cm. There was no correlation between the muscle depth and shear modulus measured, measured with CFAD (r = -0.14; p = 0.557) nor by human examiner on a relaxed (r = 0.29; p = 0.233) and stretched muscle (r = 0.310; p = 0.196).

Muscle stiffness measurements on meat specimens were shown to have excellent repeatability across repetitions when measured with the CFAD (ICCs = 0.95), across repetitions performed by single examiner (ICCs = 0.93), and moderate reproducibility between the three examiners (ICCs = 0.71). Changing the application force of CFAD resulted in low reproducibility (ICCs = 0.43). However, there was no difference in mean shear modulus between the two force application levels (F = 0.52; p = 0.481). Lowest MDC (11.3%) were present for measurements with CFAD, using constant application force. On the other hand, changing the application force resulted in MDC greater than 30%. Detailed results from the meat specimen experiment are presented in Table 2.

**Table 2:** Repeatability and reproducibility results for measurements on meat specimens.

	Trial 1 (kPa)	Trial 2 (kPa)	Trial 3 (kPa)		Repeatability and reproducibility				ANOVA	
	M (SD)	M (SD)	M (SD)	ICCs (95%CI)	ICCa (95%CI)	SEM	MDC(%)	CV(%)	F	p
CFAD (Constant force)	56.63 (14.10)	57.06 (12.02)	55.79 (11.54)	0.95 (0.88–0.97)	0.98 (0.96–0.99)	4.11	11.38	7.27	0.96	0.39
CFAD (Changed force)	56.49 (12.37)	59.02 (16.55)	/	0.43 (0.01–0.72)	0.60 (0.01–0.84)	13.06	36.19	22.61	0.52	0.48
Intra-rater (Rater 1)	61.31 (17.10)	60.35 (17.95)	59.17 (15.22)	0.93 (0.85–0.96)	0.98 (0.94–0.99)	6.20	17.19	10.29	1.15	0.33
Inter-rater (Raters 1–3)	60.27 (16.40)	58.35 (11.84)	58.99 (14.02)	0.71 (0.49–0.86)	0.88 (0.74–0.94)	9.90	27.45	16.73	0.33	0.77

M = mean; SD = standard deviation; ICCs = two-way mixed single intra-class correlation coefficient; ICCa = two-way mixed average intra-class correlation coefficient; CI = confidence interval; SEM = standard error of measurement; MDC = minimal detectable change; CV = coefficient of variance

On a relaxed BF muscle, there was good repeatability when the measurements were performed with CFAD (ICCs = 0.73) and high for a human examiner (ICCs = 0.85) Inter-rater reproducibility was also good (ICC = 0.74). Inter-visit reproducibility of measurements on relaxed BF muscles was poor, both for the trials performed with CFAD (ICCs = 0.45) and for trials performed by human examiner (ICCs = 0.34). In the relaxed BF position, there was also a statistically significant systematic error in scores measured with CFAD between visits (F = 6.89; p = 0.020).

**Fig 3:**
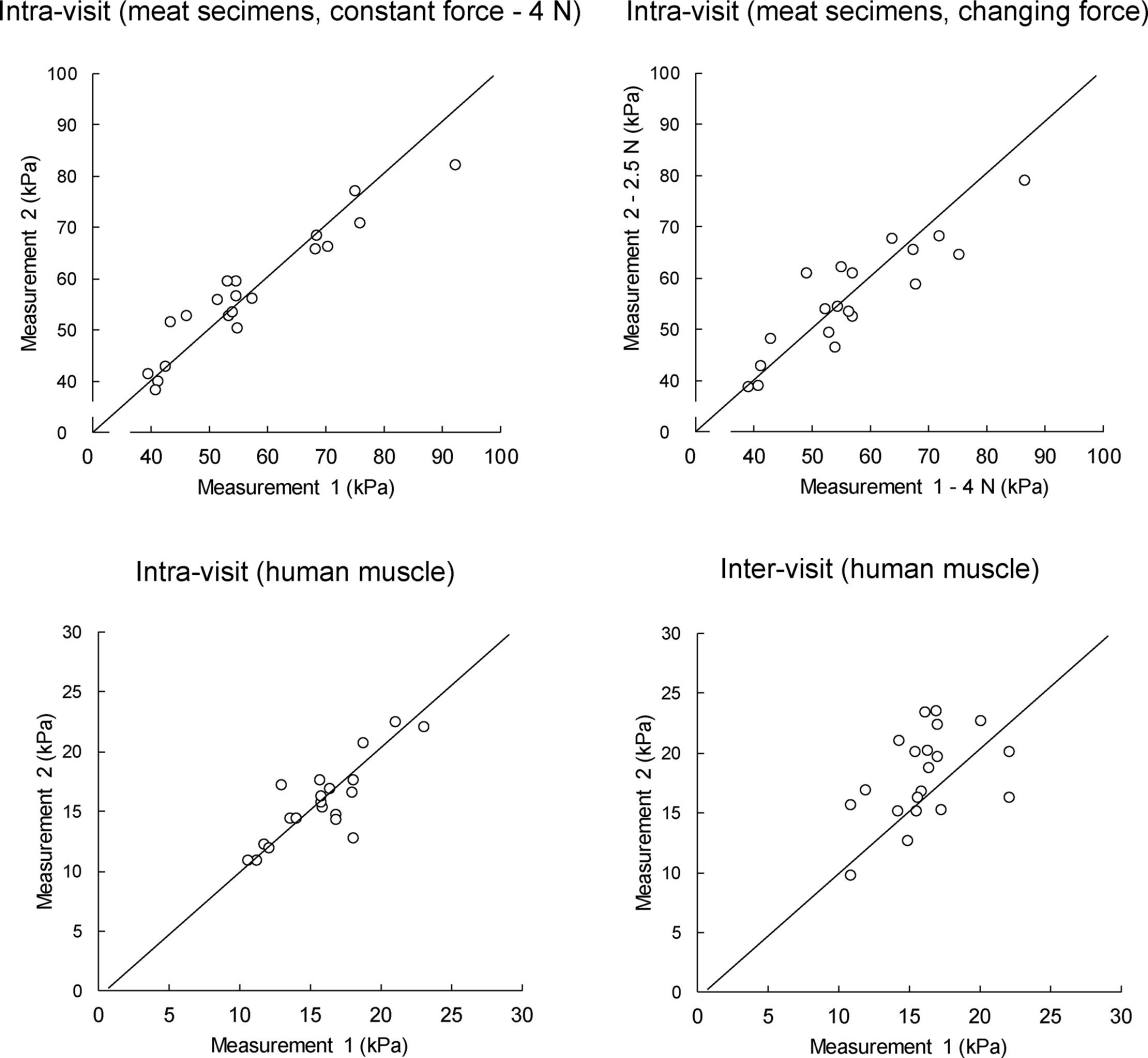
Scatter plots of measurements conducted with custom-made device. Diagonal line represents a perfect match between two measurements.

**Fig 4:**
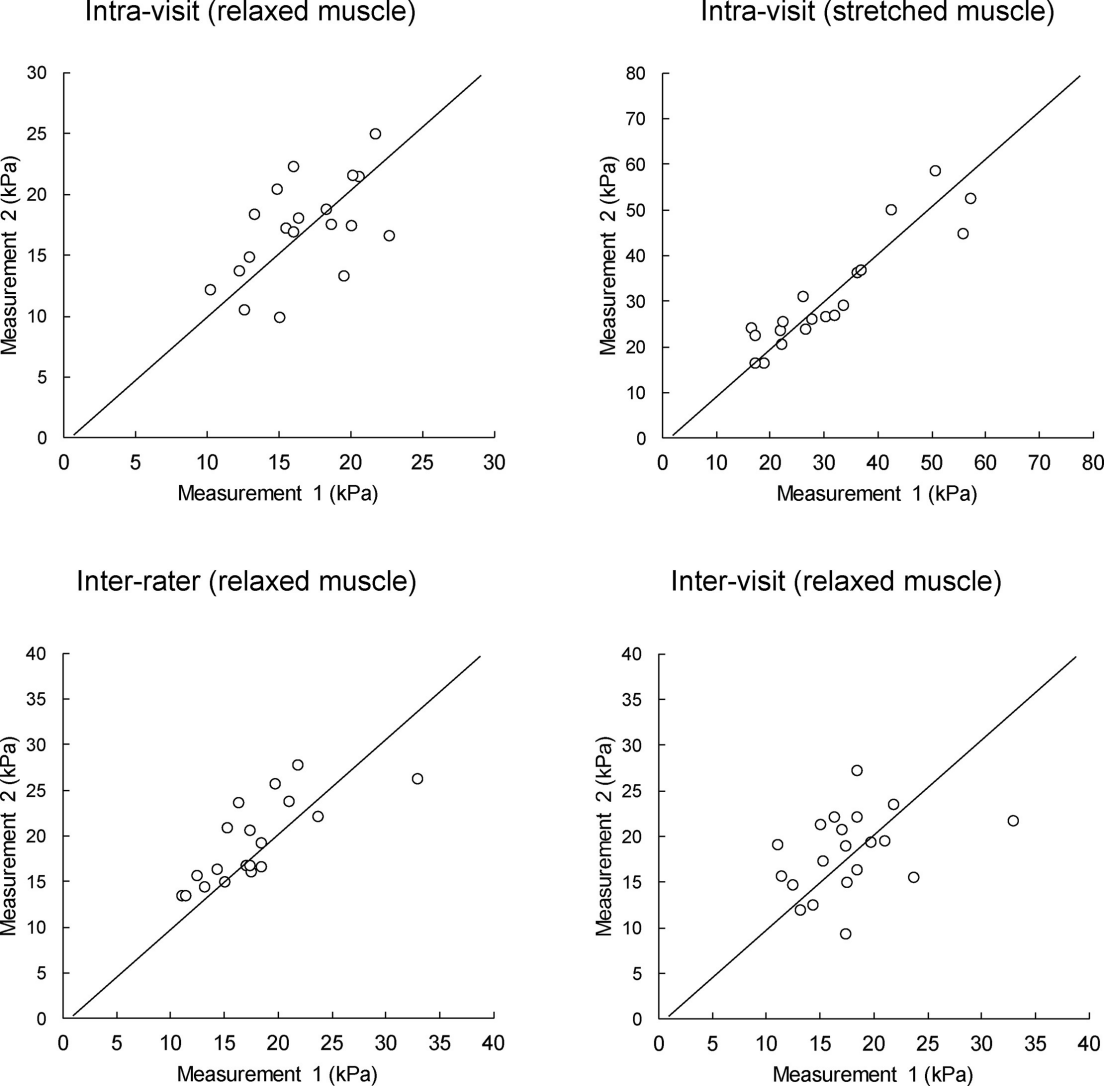
Scatter plots of measurements conducted by human examiners. Diagonal line represents a perfect match between two measurements.

**Fig 5:**
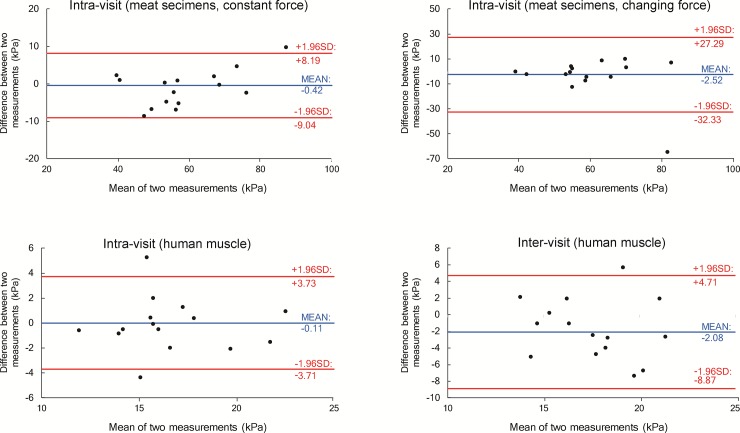
Bland-Altman plots of measurements conducted with custom-made device. Horizontal lines indicate the mean difference between measurements (blue) and 95% confidence intervals (red).

**Fig 6:**
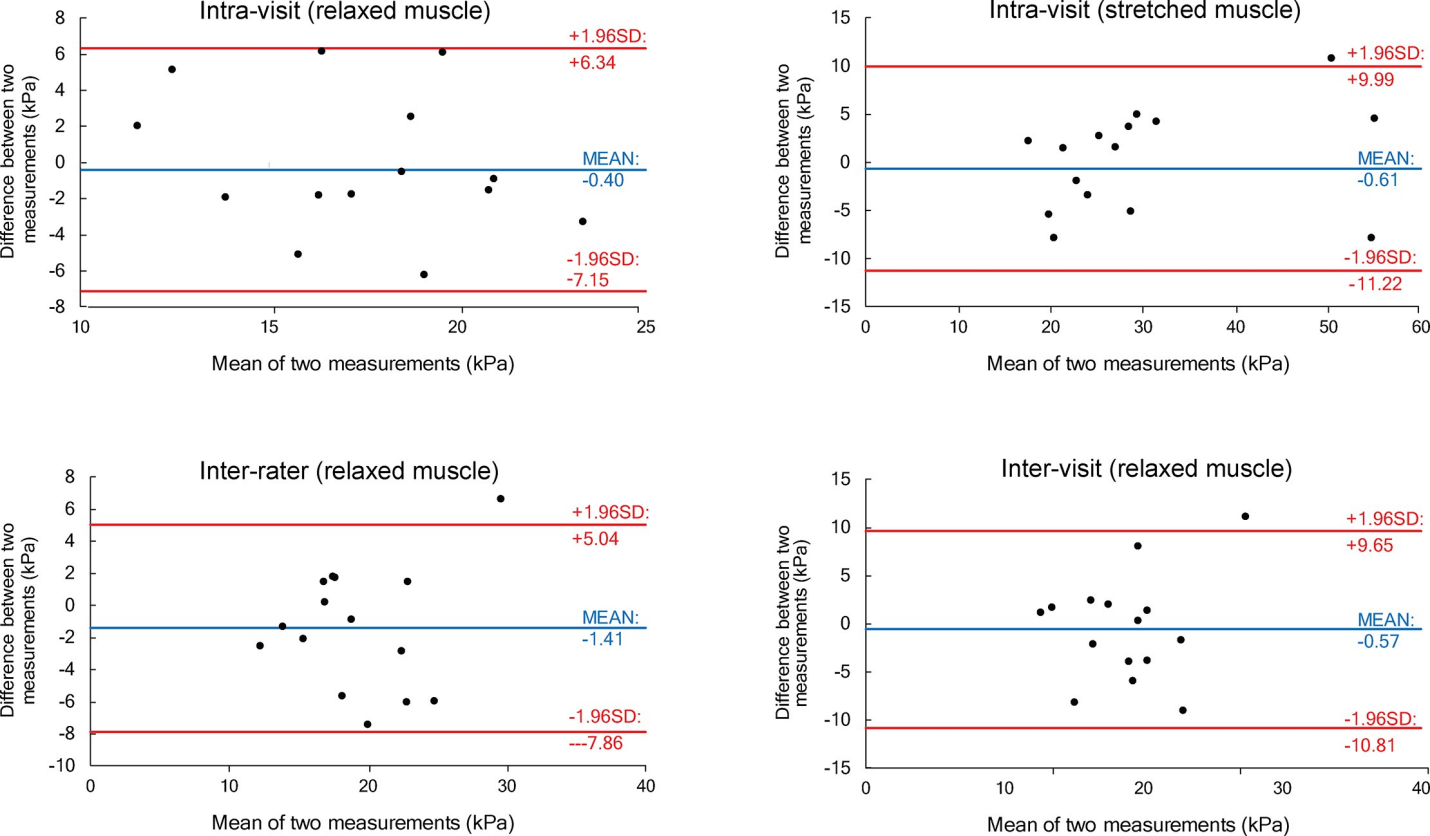
Bland-Altman plots of measurements conducted by human examiners. Horizontal lines indicate the mean difference between measurements (blue) and 95% confidence intervals (red).

For the measurement on stretched BF muscles, intra-rater repeatability (ICCs = 0.90) and inter-rater (ICCs = 0.88) and inter-visit (ICCs = 0.87) reproducibility scores were all high to excellent. Detailed repeatability and reproducibility results from the human experiment are presented in Table 2. The shear modulus in relaxed BF muscles was statistically significantly (t = 4.22; p < 0.001) lower (17.59 ± 4.91 kPa) compared to stretched BF muscles (32.58 ± 15.06 kPa). MDC were generally lower (< 10%) for relaxed muscles because of the larger between-subject variability in the stretched position (Table 3). Fig 3 and Fig 4 display the scatter plots for the measurements performed with the CFAD and by the human examiners, respectively. Additionally, Bland-Altman plots for the same measurements are provided in Fig 5 for CFAD and Fig 6 for human examiners.

**Table 3:** Repeatability and reproducibility results for measurements on human participants’ hamstring muscles.

	Trial 1 (kPa)	Trial 2 (kPa)	Trial 3 (kPa)		Repeatability and reproducibility				ANOVA	
	M (SD)	M (SD)	M (SD)	ICCs (95%CI)	ICCa (95%CI)	SEM	MDC(%)	CV(%)	F	p
		**MUSCLE RELAXED**								
Intra-visit (CFAD)	15.74 (3.25)	15.73 (3.33)	16.46 (3.41)	0.73 (0.52–0.87)	0.89 (0.77–0.95)	2.27	6.30	14.22	1.19	0.32
Intra-rater (Rater 1)	17.49 (5.18)	17.89 (5.28)	17.40 (5.09)	0.85 (0.71–0.93)	0.94 (0.88–0.97)	2.76	7.64	15.66	0.33	0.72
Inter-rater (Rater 1–2)	17.60 (4.91)	19.01 (4.48)	/	0.74 (0.45–0.88)	0.85 (0.62–0.94)	3.15	8.74	17.23	3.47	0.07
Inter-visit (CFAD)	15.98 (3.02)	18.06 (3.71)	/	0.45 (0.02–0.74)	0.62 (0.03–0.84)	3.01	8.34	17.67	6.89	0.02
Inter-visit (Rater 1)	17.60 (4.91)	18.17 (4.39)	/	0.34 (0.11–0.67)	0.51 (0.25–0.80)	4.38	12.13	24.47	0.23	0.64
		**MUSCLE STRETCHED (60% RoM)**								
Intra-rater (Rater 1)	32.58 (14.21)	33.20 (15.21)	31.95 (17.35)	0.90 (0.79–0.95)	0.96 (0.92–0.98)	6.95	19.27	21.34	0.30	0.75
Inter-rater (Rater 1–2)	32.58 (15.07)	35.02 (13.54)	/	0.88 (0.72–0.95)	0.94 (0.84–0.97)	6.74	18.68	19.94	2.62	0.12
Inter-visit (Rater 1)	32.58 (15.07)	34.25 (13.69)	/	0.87 (0.70–0.94)	0.93 (0.82–0.97)	7.09	19.65	21.22	1.03	0.32

M = mean; SD = standard deviation; ICCs = two-way mixed single intra-class correlation coefficient; ICCa = two-way mixed average intra-class correlation coefficient; CI = confidence interval; SEM = standard error of measurement; MDC = minimal detectable change; CV = coefficient of variance

## Discussion

The purpose of this study was to assess intra-rater repeatability and inter-rater and inter-visit reproducibility of SWE based assessment of BF muscle stiffness. Separate experiments were conducted on porcine meat specimens and human participants to better reveal the sources of potential errors. For the same purpose, a custom-made device was built that enabled us to perform multiple repetitions on the fixed spot and applying different levels of force. This way, the examiners’ inaccuracies in probe positioning as a potential source of error were eliminated. Furthermore, neutral and stretched position of the BF muscle were assessed separately. The results of our study showed the repeatability of SWE measurements on meat specimens to be moderate to high. Measurements on human participants showed good or high repeatability and reproducibility with small MDC values for relaxed muscles, and high repeatability and reproducibility with a higher MDC values for stretched muscles.

To our knowledge, only one study to date has examined the SWE’s validity, repeatability and reproducibility on meat specimens [13]. The authors reported a linear relationship between SWE scores and muscle tension. However, this study used fresh meat specimens that were examined no later than five hours after animal sacrifice and used a different muscle group (brachialis). Therefore, the comparability of the mean values is limited. Mean shear modulus values from our meat specimen measurement ranged from 55.79 ± 11.54 kPa to 61.31 ± 17.10 kPa, which is substantially higher than typical values for relaxed (15–18 kPa) and stretched human (31-35 kPa) hamstring muscle we obtained. Since our specimens were obtained from a commercial seller and were on average ~12 hours old, the effects of rigor mortis could have significantly increased muscles stiffness. Furthermore, lower tissue temperature is also associated with increased stiffness [35]. However, the main rationale for performing measurements on a meat specimen was to separately examine the repeatability and reproducibility of the SWE on biologically stable tissue. When measured with CFAD that enabled constant force and position of application, the repeatability of the SWE was almost perfect (ICCs = 0.95). Intra-rater repeatability was not noticeably lower (ICCs = 0.93), however, inter-rater reproducibility (ICCs = 0.71) was, indicating that probe positioning accuracy is important. Moreover, using different probe application force levels resulted in low reproducibility (ICCs = 0.43), which indicates that force should be kept as constant as possible throughout measurement. Very high repeatability with constant force indicated, that little error can be attributed to the ultrasound device itself.

In the second part of the experiment, we found good or high repeatability (ICCs = 0.73 measured with CFAD; ICCs = 0.85 measured by human examiner) and inter-rater reproducibility (ICCs = 0.74), but poor intra-visit reproducibility (ICCs = 0.34–0.45) on a relaxed hamstring muscle. It is possible that the lower repeatability of measurements done with CFAD compared to the examiner occurred due to the small movements of the participant’s legs, which the examiner (but not CFAD) could have adjusted for. This is supported by the fact that the repeatability of the measurements done with CFAD was higher than the repeatability of the examiner when measurements were performed on completely stable meat specimens. All aspects of reliability were high in the stretched muscle (ICCs = 0.87-0.90). This phenomenon is in accordance with the results of a previous study [20], who found higher ICC scores when the hamstrings were stretched to 60% of range of motion, and lower or similar scores when the stretch was extended to 90% of range of motion. They measured all hamstring muscles separately, and their intra-rater repeatability scores for all of those (ICCs = 0.71–0.94) were similar to the results obtained in the present study (ICCs = 0.85–0.90). Despite the higher ICC scores in a stretched BF position, MDC were lower (< 10%) for relaxed muscles because of the larger between-subject variability in the stretched position. The intra-rater MDC value on relaxed BF muscles (7.6%) is low compared to the magnitude of change reported for acute effects of static stretching on BF muscle shear modulus (i.e. -23%) [15]. The inter-visit MDC for relaxed BF muscles was also lower (12.1%) than the changes reported to be induced by a 4-week static stretching programme (i.e., 14%) [36]. These findings suggest that SWE measurements on relaxed muscles are sensitive enough to reliably detect typical changes expected to follow from acute and long-term interventions. In contrast, all MDC values for stretched BF muscles were higher (18–19%). Therefore, it seems that BF muscle stiffness assessment with SWE should be performed on a relaxed muscle to increase the confidence of the measurements, despite higher ICC values in the stretched position.

Comparably high repeatability and reproducibility scores for assessing muscle stiffness with SWE were previously reported for deltoid muscle [29], patellar tendon and rectus femoris [31], and trunk muscles during contraction [32] and at rest [30]. The latter study also demonstrated the importance of the examiner’s skills in ultrasound imaging (e.g. intra-visit ICCs = 0.86 for skilled examiners; ICCs = 0.59 for unskilled examiners). Examiners in our study had been trained briefly (2 sessions of 1 hour) by an ultrasound expert, but were not experts in ultrasound imaging themselves. It is possible that the repeatability and reproducibility of SWE imaging on hamstring muscles are higher when performed by highly skilled ultrasound operators.

As expected, mean shear modulus increased in the stretched muscle position. Previous studies have documented that shear modulus is increased along with the passive muscle tension [21], but decreases below the baseline values when the tension is removed [23]. In our study, we lowered the leg to a neutral position between repetitions to avoid an acute effect of the passive tension applied. However, a certain level of effect between repetitions cannot be ruled out. For the examiner whose scores were used to calculate intra-rater reproducibility, no statistically significant differences (F = 0.296; p = 0.745) across repetitions were found.

In conclusion, we demonstrated that evaluation of BF muscle stiffness using SWE is reliable, with a lower MDC for a relaxed muscle position as compared to a stretched muscle position. Practitioners need to be aware that keeping application force consistent and site of application as constant as possible will likely increase the repeatability of their measurements. Examiners without ultrasound imaging experience should undergo a sufficient amount of training before performing SWE measurements. With the above in mind, SWE imaging could serve as an important tool for assessing BF muscle stiffness, particularly (but not limited to) in interventional studies investigating ways to prevent hamstring injuries or improve sport performance.
